# The Influence of Sociodemographic Factors on Mobile Device Use among Young Children in Putrajaya, Malaysia

**DOI:** 10.3390/children9020228

**Published:** 2022-02-08

**Authors:** Nur Nabilah Abdullah, Suziyani Mohamed, Kamariah Abu Bakar, Noratiqah Satari

**Affiliations:** 1 Faculty of Education, Universiti Kebangsaan Malaysia, Bangi 43600, Malaysia; nurnabilah414@gmail.com; 2 Centre of Education and Community Well-Being, Faculty of Education, Universiti Kebangsaan Malaysia, Bangi 43600, Malaysia; kamariah_abubakar@ukm.edu.my; 3 Faculty of Human Development, Universiti Pendidikan Sultan Idris, Tanjong Malim 34500, Malaysia; atiqahsatari@upsi.edu.my

**Keywords:** mobile device, young children, gadget

## Abstract

Technology is evolving rapidly around the world, and the use of mobile devices is increasing every day. Today, everyone owns a mobile device, including young children. Parents provide and allow young children to use mobile devices for various purposes. Due to the fact of these circumstances, children begin to become comfortable with the use of mobile devices, and they are prone to excessive use. Therefore, the purpose of this study was to examine the influence of sociodemographic factors on excessive mobile device use among young children. Sociodemographic variables, including the child’s gender, the child’s age when starting to use a mobile device, the parent’s educational level, household income, type of application used, and the purpose of giving a mobile device to the child, were selected as predictive factors. A cross-sectional survey study design with a quantitative approach was conducted. A simple random sampling technique was employed, and a total of 364 parents completed the adapted questionnaire, namely, the Problematic Mobile Phone Use Scale (PMPUS). Data were statistically analyzed using descriptive and binary logistic regression analysis. The findings revealed that gender, age of the child when starting to use mobile devices, and purpose of parents providing mobile devices significantly contributed to 77.7% of the variance to make children users with a problem. However, the parent’s educational level, household income, and type of application did not significantly contribute to the problem of mobile device use. Later, this study discusses the research implication, limitation, and recommendation for future research based on the finding.

## 1. Introduction

Mobile devices with internet access are a vital tool for every individual regardless of social background due their functions that can facilitate daily life. By using a mobile device, tasks can be managed from home without having to meet others. This situation leads to changes in the way humans communicate, interact, and accomplish daily routines. As a result, human contact is diminishing as the opportunity to interact face to face is also reduced [1,2]. Today, everyone uses mobile devices for various purposes such as gaming, web surfing, social media, and online chats including children [3]. The use of mobile devices among young children is increasing every year [4,5]. This scenario had raised awareness among experts about the negative impact of the excessive use of mobile devices on children’s development. Furthermore, young children are exposed to online activities and mobile device content [6,7,8,9].

It is suggested that the use of mobile devices by children be properly monitored by parents to avoid excessive use. Parents should implement strict rules in controlling the duration and purpose of use [10,11]. Today, children prefer to sit alone and preoccupy themselves with activities on mobile devices. They tend to spend more time interacting with digital screens rather than playing, interacting face to face, and being active [10,12]. If this scenario is not handled carefully, children may forget the fun of playing with family and friends. Meanwhile, play has proven to be effective in stimulating children’s development across domains. Furthermore, poor quality time spent with family members and friends causes weakened family bonds [10,13,14], contributing to behavioral problems [15,16] and poor social–emotional competence [17,18], social communication, and social interaction skills [15,18,19,20].

Interpersonal skills involve children’s communication with others, which should involve eye communication, body position and movements, facial gestures and expressions, voice intonation, language, and listener interaction [21]. Scholars have emphasized the negative impact of mobile devices on children’s interpersonal skills [22] as they lack opportunities to socialize with peers and family. Consequently, children have difficulty conveying the information that is in their minds. Without socializing, children cannot practice communication and interaction skills. Children are reported to have difficulty with eye contact, gestures, facial expressions, and voice intonation [23]. This, when prolonged, can cause children’s communication process to be stunted at an early age, which, in turn, invites verbal skill problems [20,24].

A previous study found that there was a significant relationship between the duration of mobile device use with psychosocial effects and children’s interaction with peers in preschool [25]. Children who use mobile devices for a longer period are often associated with behavioral problems in self-regulation [26] and have attention problems [27,28]. These children are prone to unstable emotional outbursts, exhibit impulsive behavior, experience emotional disturbances, become aggressive and misbehave [24], and have limited interaction with peers at school [25,29].

Moreover, excessive use of mobile devices also contributes to poor social–emotional competence among young children due to the poorly nurtured stimuli [18,30]. Furthermore, online games encourage children to be individualistic and more likely to be selfish and less cooperative. If such personalities are adapted in real life, then children are less tolerant in society. They will also find it difficult to accept defeat or failure if participating in a competition and will try to dominate the competition. Children who are overly dependent on mobile devices are more likely to be angry, restless, and uncomfortable when they are unable to access the devices [31,32]. In addition, excessive mobile device use leads to a lack of prosocial behaviors in young children. Children are reported to have difficulty with daily interactions, cooperative skills, lack of empathy, and not helping others [15,27].

Other than negative effects, the proper use of mobile devices and technology will lead to positive outcomes that benefit children. Some studies stated the positive effects of mobile device use on educational attainment [30,33,34,35,36]. The use of mobile devices in the classroom can help attract and engage children in learning activities [30,34]. In addition, mobile devices help enhance children’s learning motivation [35] and promote active involvement in the classroom [36,37]. In addition, mobile device use provides a positive effect in promoting healthy social–emotional development among young children [38].

Despite the positive and negative effects of mobile device use on young children’s development, it is vital to identify the predictive factors that contribute to this situation. In Malaysia, there has been limited research conducted on mobile device use among young children [39,40], especially on identifying predictive factors. The existing research focuses more on the impact of mobile device use on young children’s communication [39], social–emotional development [38,39,40], physical activity [41], and eye health [42,43]. Therefore, this study aimed to examine the predictive factors that contribute to excessive mobile device use among young children.

## 2. Literature Review and Hypothesis Development

This research aimed to determine the contribution of the predictive factors to excessive mobile device use among young children. Sociodemographic variables, including the child’s gender, the child’s age when starting to use a mobile device, the parent’s educational level, household income, type of applications used, and the purpose of giving a mobile device to the child, were selected as predictive factors. These variables were selected based on previous studies [3,21,32,44,45,46,47].

Several studies reported a significant positive association between girls and problematic mobile device usage [48,49,50,51,52,53,54,55]. Contrasting this, several studies documented a significant positive association between boys and problematic mobile device usage [56] or revealed higher scores in boys than in girls [32,44,45,57]. Yet, some studies show gender is not a predictive factor of problematic use of mobile devices [3,58,59]. Girls and boys have different preferences for mobile device activities [48]. Girls likely spend time on learning activities [31], communication, and social networking applications [44,60]. Meanwhile, boys are keener on video gaming and television viewing [44,48].

The parent’s educational level significantly contributes to problematic mobile device use as reported in [53,58,61,62]. Parents with lower educational levels tend to allow their children to spend extra time on a mobile device compared to parents with higher educational levels [22,62,63,64,65]. Parents with higher educational levels are more aware and tend to guide their children on the appropriate period of use [63,66] and suitable apps and websites to access [64,67]. Contrary to this, several studies reported there was no effect of the parent’s educational level on problematic mobile device use [44,58].

Household income is a crucial aspect in influencing an individual’s life, as well as young children. There are many things that parents can provide to their children with money, including personal mobile devices and internet access. Having a mobile device makes it difficult for parents to monitor usage, which, in turn, contributes to excessive use. Because of this, many studies have reported that children with high household incomes are prone to problematic mobile device use [68,69,70,71]. However, [44,58] reported that there was no effect of household income on problematic mobile device use.

Existing studies have reported that there is a significant relationship between frequent types of application usage with excessive mobile device use. Children that used a mobile device for entertainment purposes, such as for playing games [11,72,73,74], viewing television [75], and communication [76,77], were more likely to be problematic users. Meanwhile, children that used a mobile device for educational-related purposes were not problematic users [74]. However, there are limited studies examining the relationship between children’s ages when they start using a mobile device, the purpose of giving mobile devices to children, and problematic mobile device use.

The Malaysian National Population and Family Development Board [78] reported that 78.3% of parents in Klang Valley allow their children to own mobile devices. In addition, the report stated that 50.1% of those children spend more than three hours per day on a mobile device, and 29% are problematic users. Even more worrying is that parents provide and allow children to use mobile devices to keep them inactive for parents to easily conduct housework, to calm children whenever they are throwing tantrums, and to control children’s behavior if they are in someone else’s house or in a restaurant.

Considering the inconsistent findings and limited data from previous research, the predictive factors of excessive mobile device use in Malaysia are questionable. Thus, the predictive factors for the Malaysian population may be different from those reported in other populations. Therefore, the following hypothesis was proposed in this study:

**Hypothesis** **1** **(H1):** *Sociodemographic factors significantly contribute to excessive mobile device use among young children*.

## 3. Materials and Methods

### 3.1. Study Design

This study employed a cross-sectional survey study design with a quantitative approach. The design and approach of this research were chosen as data could be obtained quickly for a large study sample size.

### 3.2. Participants

A total of 364 respondents participated in this study. A total of 51.6% (*n* = 188) of the children were girls, and 48.4% (*n* = 176) were boys. In terms of age, 51.1% (*n* = 186) of the children were 5 years old, and 48.9% (*n* = 178) were 6 years old. In addition, approximately 53.3% (*n* = 194) of parents were aged 36 years and above, 45.9% (*n* = 167) were aged between 26 and 35 years, and 0.8% (*n* = 3) were aged 25 years and below. Regarding the level of education, 35.7% (*n* = 130) of parents had a bachelor’s degree, 25.0% (*n* = 91) had a certificate, 21.4% (*n* = 78) had a diploma, and 17.9% (*n* = 65) had a postgraduate degree. In this study, a certificate refers to a person with a Malaysian Certificate of Education (SPM). In Malaysia, citizens will sit for the Malaysian Certificate of Education examination in the final year of high school. A citizen who fulfills the SPM requirements will be awarded a certification. Meanwhile, a diploma refers to the tertiary education program attended by citizens after receiving an SPM. The duration for a diploma education is three years.

Furthermore, information on household income was also collected to ensure the child participants for this study came from each economic group. In Malaysia, economic status is divided into three categories, namely, the Top 20 (T20), Middle 40 (M40), and Bottom 40 (B40) [79]. T20 refers to Malaysian citizens earning more than MYR 9620 per month (approximately USD 2287.86). This group makes up 20% of the population in Malaysia. M40 refers to a middle-class group with a monthly household income ranging from MYR 4360 to MYR 9616 (approximately USD 1036.91–2286.91). This group makes up 40% of the population in Malaysia. Meanwhile, B40 refers to a group of citizens earning less than MYR 4360 for a monthly (approximately USD 1036.91) household income. This group makes up 40% of the population in Malaysia. Approximately 36.0% (*n* = 131) of the children came from the M40 group, 33.2% (*n* = 121) from the T20 group, and 30.8% (*n* = 112) from the B40 group. Table 1 presents the demographic information on the children and their families.

An analysis was performed to gather information on mobile device use by children. In this study, 72.8% (*n* = 265) of the children were smartphone users, and 27.2% (*n* = 99) of the children were tablet users. Approximately 83.2% (*n* = 303) of the children started using a mobile device at the age of 3 years old and below, and 16.8% (*n* = 61) started at the age of 4–6 years old. Regarding the types of application, 80.5% (*n* = 293) of the children used mobile devices for entertainment purposes such as playing games, web surfing, listening to music, or watching videos. Meanwhile, 19.5% (*n* = 71) of children used mobile devices for educational-related purposes. Analysis of the item, namely, mobile device ownership, showed that 78.8% (*n* = 287) of the children used their parents’ devices, 11.8% (*n* = 43) of the children used other family members’ devices, and only 9.3% (*n* = 34) of the children had their own devices. Parents were also asked about the purposes of providing mobile devices to their children. Approximately 34.1% (*n* = 124) of parents allowed them to use it for educational purposes, 32.7% (*n* = 119) of parents responded that they allowed them to use it to make their child sit still, 22.5% (*n* = 82) of parents allowed their children to use it to be up-to-date with technology, and 10.7% (*n* = 39) of parents provided mobile devices to avoid and to calm down tantrums. Table 2 presents detailed information on the uses of mobile devices.

### 3.3. Procedure

Before the data collection process, an application to conduct a study was made to the ethics committee from the Faculty of Education, Universiti Kebangsaan, Malaysia. The approval was received on 9 October 2019. Later, a letter of permission to distribute the questionnaire was given to the owner of the early childhood center. This study was conducted in Putrajaya, Malaysia, due to the diverse backgrounds of the population in terms of education levels and household income. According to data from the Department of Statistics Malaysia, there were 6200 children aged five to six years residing in Putrajaya in 2020. The sample size was estimated using a table from [80]. Based on the table, the actual sample size for this study was 364. A simple random sampling technique was employed in the data collection process. Questionnaires together with an information consent letter were distributed to parents through the school, and a total of 364 questionnaires were collected with a 100% return rate.

### 3.4. Instrument

This study used a questionnaire for the data collection process. The questionnaire consisted of two sections, namely, demographic information of the participants and the mobile devices use scale. The items for the mobile device use scale sections were adapted from previous research that used the Problematic Mobile Phone Use Scale (PMPUS) [81]. The original instrument consisted of four subdimensions with 26 items and was distributed to university students. The four subdimensions were deprivation, adverse outcomes, control problems, and interaction avoidance. Deprivation evaluates feelings such as anxiousness or uneasiness when the mobile device is not available or not in a usable state; adverse outcomes assess the negative effect of mobile device use on an individual’s daily life; control problems measure an individual’s ability to control their use of mobile devices; interaction avoidance determines the communication preferences either via online or face-to-face interactions [81]. The original instrument consisted of eight items that measure deprivation (labeled D1, D2, D3, D4, D5, D6, D7 and D8), seven items that measure adverse outcomes (labeled AO9, AO10, AO11, AO12, AO13, AO14 and AO15), six items that measure control problems (labeled as CP16, CP17, CP18, CP19, CP20, and CP21), and five items that measure interaction avoidance (labeled as IA22, IA23, IA24, IA25 and IA26). The items were measured using a five-point Likert-type scale, and the response options were: 1 = not appropriate at all; 2 = rarely appropriate; 3 = somewhat appropriate; 4 = fairly appropriate; 5 = completely appropriate.

Cross-cultural adaptation guidelines were applied by taking into account the translation process and cultural appropriateness [82,83]. The translation process aimed to ensure that the instrument was acceptable and relevant for use by the Malaysian population [84,85,86]. The instrument was translated into the Malay language by a Malay–English speaker. Then, two linguists examined the semantic equivalence of the instrument between the original version and the Malaysian version. Later, a back-translation procedure was performed by a Malay–English speaker who did not know the original version of the instrument. The back-translation process took two weeks. The development of this instrument is explained in Section 3.4.1 and Section 3.4.2, and the list of adapted items is outlined in Appendix A.

#### 3.4.1. Validity and Reliability

Three experts in early childhood education, psychology, and family counseling evaluated the content validity, culture, and age appropriateness of the adapted version of the PMPUS. A focus group discussion session was conducted with all experts, and they recommended that eight items be removed due to the fact of issues such as irrelevance and repetition. Five items were identified as not appropriate for use with children aged five-to-six years. Moreover, the experts also agreed that it would be difficult for parents to observe the following behavior in children: “my child feels insecure without a mobile device” (D4); “my child will feel lonely without a mobile device” (D7); “my child will feel lost without a mobile device” (D8); “my child is always checking mobile devices” (CP18); “my child wants to use a mobile device again immediately after they stop it” (CP21). Item IA26 (i.e., “my child would rather make friends through social media than real life”) was also removed. Experts pointed out that the majority of children in Malaysia aged five to six still do not own social media accounts.

Furthermore, experts also suggested that two items be removed due to the fact of repetition. One of the items was “my child is too busy using a mobile device, which interferes with his daily routine” (AO9). The experts agreed that this question was also asked in AO10 to AO13, which asked about children’s mealtimes, sleep habits, and learning activities, referring to their daily routine. The second item removed was “my child is busy using a mobile device so that he is less sociable with the people around him” (AO15). According to experts, this item has the same meaning as the item “my child would rather spend time with a mobile device than hang out with people around him” (AO14). Both items measured children’s social interactions and communication with people around them. In addition, the experts also commented on the sentence structure so as to enhance comprehension and clarity. All comments were taken into account to improve the quality of the items. Then, the content validity index (CVI) was calculated using the formula in [87]. The CVI value for this instrument was 0.90. According to [88], a CVI ≥ 0.80 is acceptable.

Initially, the adapted version of the PMPUS consisted of 18 items with a five-point Likert scale and the responses 1 = never, 2 = rarely, 3 = sometimes, 4 = very often, and 5 = always. The Likert scale responses were altered to ensure they were appropriate for the items and participants completing the questionnaire. Compared to the original version, which could be completed by the respondents themselves, this adapted version was prepared to be completed by the parents. Moreover, it was impossible to distribute questionnaires to children aged five-to-six years due to the fact of their lack of comprehensibility. However, the constructs were retained to be the same as the original instrument. The scores were calculated based on the response from the respondent for each item. The minimal score was 18, and the maximum score was 90. An increasing score showed that the person’s level of problematic mobile device use was rising [81]. Later, total scores were calculated, and four user categories were determined based on cut-off points as performed in [21], considering the 95th, 80th, and 15th percentiles. Four categories were established, namely, casual user, regular user, at-risk user, and problematic user. Table 3 presents information on the user categories, percentiles, and scores ranges.

Item reliability measures were performed using Cronbach’s alpha. The Cronbach’s alpha values obtained ranged from 0.899 to 0.909. Overall, the Cronbach’s alpha value for the adapted version of the PMPUS was α = 0.904. This demonstrated that the adapted instrument had a good degree of internal consistency. Detailed analysis of each item showed higher scores in items CP17 “my child uses a mobile device beyond the set period” (*M* = 2.431, *SD* = 1.028) and CP20 “my child will look for a mobile device as soon as he wakes up” (*M* = 2.586, *SD* = 1.026). Table 4 presents the mean, standard deviation (*SD*), and Cronbach’s alpha values of the items.

#### 3.4.2. Demographic Information

The demographic information of the respondents consisted of three sub-sections, namely, child characteristics, family characteristics, and mobile device information. A total of ten items were asked based on three sub-sections: child’s gender, child’s age, parent’s age, household income, parent’s level of education, types of mobile devices, child’s age at the start of mobile device usage, frequent types of applications used, and the purpose of the mobile device’s provision.

### 3.5. Analysis

The analysis was conducted using SPPS^®^ version 26.0 for Windows™ (IBM Corporation, New York, NY, USA). Descriptive statistical analysis was performed to collect information on the frequency, mean, standard deviation, and mode. Inferential statistical analysis used binary logistic regression to examine the influence of demographic variables, namely, gender, the child’s age when starting to use a mobile device, parent’s level of education, household income, types of mobile devices, types of applications, and the purpose of providing mobile devices to the child. The four categories of mobile device users were transformed into two groups to enable binary logistic regression to be carried out as suggested in [32,45]. This was performed by considering the sum of the at-risk users and problematic users as users with a problem. Meanwhile, the sum of the casual users and regular users was considered as users without a problem. Before data analysis, a data-screening process was conducted to ensure the data were clean and free from errors [89].

## 4. Results

### 4.1. Exploratory Factor Analysis (EFA)

Exploratory factor analysis was employed, and the principal axis factoring method with varimax rotation was performed to compute the underlying structure of the 18 items of the adapted instrument. Bartlett’s test of sphericity (χ^2^ = 3023.030, *p* = 0.000) indicated that the variables were correlated highly enough to provide a reasonable basis for factor analysis. Meanwhile, the Kaiser–Meyer–Olkin measure of 0.909 indicated adequate items for each factor. Four factors were studied based on the number of the constructs, namely, deprivation (Factor 1), adverse outcomes (Factor 2), interaction avoidance (Factor 3), and control problems (Factor 4). After rotation, the first factor accounted for 16.46% of the variance, the second factor accounted for 14.50%, the third factor accounted for 13.11%, and the fourth factor accounted for 9.60%. Table 5 presents the items and factor loadings for the rotated factors, with a loading less than 0.30 excluded to improve clarity.

### 4.2. User Categories

The result showed that 64.3% (*n* = 234) of the children were regular users with an average score of 35.46 (*SD* = 5.33). In addition, 17.6% (*n* = 64) of the children were at-risk users with an average score of 48.59 (*SD* = 2.34). Moreover, 14.8% (*n* = 54) of the children were casual users with an average score of 21.52 (*SD* = 2.13). Problematic users were represented by 3.3% (*n* = 12) of the children with an average score of 59.50 (*SD* = 2.72). Table 6 presents detailed information on the analysis of user categories.

### 4.3. Predictive Sociodemographic Factors of Mobile Device Use

The four categories of mobile devices users were transformed into two groups, namely, users without a problem and users with a problem. This was performed by joining the existing categories. The at-risk users and problematic users combined to form a group of users with a problem (20.9%). Meanwhile, casual users and regular users combined to form a group of users without a problem (79.1%). Binary logistic regression was performed to assess the effect of gender, child’s age when starting to use a mobile device, parent’s level of education, household income, types of mobile devices, types of applications, and the purposes of providing mobile devices to the child on the likelihood that children become users with a problem.

The model containing all predictors was statistically significant χ^2^ (7, *n* = 364) = 33.20, *p* = 0.002, indicating that the model was able to distinguish between users with a problem and users without a problem. The model explained between 8.7% (Cox and Snell *R* square) and 13.6% (Nagelkerke *R* squared) of the variance in mobile device use and classified 77.7% of cases correctly. The Hosmer–Lemeshow tested the null hypothesis in which the predictions made by the model fitted perfectly with the observed group membership. A Chi-square statistic was computed by comparing the observed frequencies with those expected under the linear model. The chi-square value was 3.564 with *p* = 0.894. A non-significant chi-square indicates that the data fit the model well.

As shown in Table 7, three independent variables made unique statistically significant contributions to the model, namely, gender, the child’s age at start of use of a mobile device, and the purposes of providing mobile devices to the child. Boys were 0.470 times more likely to become users with a problem than girls. Meanwhile, children introduced to mobile devices at an earlier age were more likely to become users with a problem (0.262 times). The purpose of providing mobile devices to make the children sit still was 1.142 times, which may make children become users with a problem. Based on the binary logistic analysis, the regression equation model for this study was:

Mobile Devices Users = −1.141 + (−0.755) gender + (−1.341) age when starting to use mobile device + (1.142) the purposes of providing mobile devices to the children.

## 5. Discussion

This study showed that the use of mobile devices among young children in Putrajaya may not be alarming. with the number of users for each category as follows: casual users = 14.8% (*n* = 54), regular users = 64.3% (*n* = 234), at-risk users = 17.6% (*n* = 64), and problematic users = 3.3% (*n* = 12). Meanwhile, the cumulative percentage of users without a problem was 79.1% (*n* = 288) and users with problem was 20.9% (*n* = 76). Although the percentage of users with a problem could be considered small, it should be given attention. It is possible that the percentage of users with a problem will increase without proper monitoring and guides provided.

These findings contrast with the current scenario in other countries. Scholars reported that children’s dependence on mobile devices has increased in the last three-to-four years, especially for primary school children in developed countries [90,91]. This difference may exist due to the status as a developed country, which leads to accessibility to high technology infrastructure [5], as it experienced a technological revolution earlier than developing countries such as Malaysia. It is reported that the usage of mobile devices among children below eight years old increased from 34% in 2011 to 72% in 2013 [92].

In addition, user categories were also closely related to children’s environment involving their microsystems such as parents and siblings. This study can infer that the use of mobile devices among young children in Putrajaya is still under control. Parents play a role in creating a conducive environment for the use of the mobile device by providing guidance and instruction to children. The use of mobile devices by children needs to be properly controlled by parents, especially the duration and purpose of use so that children’s development takes place holistically [91,92,93,94]. In addition, it helps children avoid using mobile devices excessively.

Children who always use a mobile device will begin to feel comfortable using it without socializing with others [95]. Therefore, this characteristic was similar to the impulsive-antisocial pathway pattern as described in the model of Mobile Phone Use Problem Patterns. This characteristic is related to individuals with mobile phone use problems. They have impulsive actions and low self-control power, which, in turn, results in the use of the device not being well-controlled.

The results of the binary logistic regression analysis showed that three out of seven independent variables of demographic factors had a significant influence on children’s mobile devices use. The three independent variables were gender, the child’s age when starting to use mobile devices, and the purpose of the parent providing a mobile device to the child. A total of 13.6% of the variance in becoming problematic users was explained by gender, age, and purpose. The remaining 86.4% contributed to other factors.

Based on the results of the analysis, gender had a significant influence on patterns of mobile device use. Boys were more likely to be problematic users with a factor of −0.755. This finding is consistent with other studies [44,46,60,96,97] that reported boys were more prone to excessive mobile device use, while several studies reported the opposite [45,74]. However, the majority of these studies were conducted on adolescent and adult populations. Boys and girls have different preferences in using mobile devices. Boys spend more time on mobile devices to play games and girls use them for social media [44,60]. These preferences explain the differences in the findings in this research. In Malaysia, children aged five to six rarely have social media accounts. Therefore, girls did not use mobile device for a longer duration than boys.

This study also found that the age of children introduced to mobile devices contributed significantly to user with problem with a factor of −1.341. Children who are exposed and allowed to use their mobile devices earlier will be susceptible to becoming users with problem. Children’s dependence on mobile devices will increase as they become more comfortable with it and less interested in making social connections with peers or family members [23]. In addition, children will see mobile devices as their source of happiness.

The purpose of parents providing mobile devices to their children to make their children sit still significantly contributed to users with problem with a factor of 1.142. Meanwhile, other purposes did not significantly contribute to users with problem. In Malaysia, it is common for parents to provide mobile devices to their children while having mealtimes at the dining table. The purpose is to make sure the children sit still while waiting for the food to be served. Some of them continue to use a mobile device while eating.

This study also showed that the parent’s level of education did not significantly contribute to the problem of mobile device use. This suggested that children have the same patterns of mobile device use despite having parents from different educational level backgrounds. This finding contradicts previous studies [69,98,99,100] that indicated that the highest level of parental education contributed to users with a problem. Putrajaya is an administrative district of the Malaysian government, and the majority of its residents are government employees. As employees, parents in this study had to use computers and technology daily. They can be classified as parents knowledgeable about technology. Therefore, they can monitor, support, and guide their children to use mobile devices and technology positively. In this modern era, everyone can find information about the advantages and disadvantages of excessive mobile device use through the internet. Knowledgeable parents can assist their children in selecting age-appropriate and developmentally appropriate applications that can benefit children [101]. Children who receive positive guidance about mobile device use tend to have positive perceptions towards the use of technology in daily life [47,62,102,103,104,105,106].

The results of binary logistic regression showed that household income did not significantly contribute to excessive mobile device use. This finding explained that children exhibit similar patterns of mobile device use regardless of their family’s economic status. This is contrary to previous research [68,70,71,107,108,109] reporting that household income significantly contributed to excessive mobile device use. Typically, high-income parents can afford to provide mobile devices together with internet access for their children. Availability and accessibility to a mobile device and the internet make children more vulnerable to excessive use of mobile devices.

However, the research findings provided a different explanation. In Malaysia, mobile devices can be purchased at low prices and also provide the facility to download applications for free with the availability of the internet. Because of this, every family can provide mobile devices for use by their children. However, an internet subscription is relatively expensive, and some families cannot afford to subscribe to Wi-Fi at home or have unlimited internet access on their mobile devices [109]. Internet access limitations cause children to only be able to access certain applications and, thus, reduce their interest in mobile devices.

### 5.1. Implications

The preventive measure should take into consideration to ensure the percentage of users with a problem is not increased. This can be achieved by taking into account the sociodemographic factors that proved to significantly contribute to the excessive mobile device use in this study. Therefore, this subtopic discusses the implications of this study. The findings of this study can be used as additional information in drafting a guideline on the mobile device used specifically for young children by the government and stakeholders. The information on the positive relationship between the child’s gender, the child’s age when starting to use a mobile device, and the purposes of giving a mobile device to the child with excessive mobile device use could be included in the guidelines. In addition, the guideline may propose a suitable age range to introduce and allow children to use the mobile device.

Then, the information on the guidelines could be conveyed to the parents, community, and early childhood educators through an awareness campaign at the national level. Thereby, parents will be more alert regarding the suitable age to introduce the mobile device to their children, did not use a mobile device as calming object whenever their children throw a tantrum, and give more attention to a boy who uses a mobile device than a girl. In addition, parents should also provide a balance of activities children’s daily so that they can grow holistically.

### 5.2. Limitations and Scope of Future Research

Even though this study was conducted with meticulous detail, some limitations still exist. Firstly, there are limitations regarding the location and sample selection. This study involves 364 participants who reside in Putrajaya. Putrajaya is the administrative center of the government of Malaysia and considered an urban area. Moreover, the majority of its residents are government servants. Thus, it is suggested that future studies involve parents and children from all states in Malaysia including suburban and rural areas, and parents from various fields of employment. Thereby, the finding could be generalised to the Malaysian population.

In addition, it is recommended that a future study take into account the race information of the participant. Malaysia is a multiracial country, and every family from a different race is practicing different norms and values in their daily life including the way of raising a child. It could be interesting to investigate the differences in mobile device practices based on race factors. A previous study from Singapore reported Malay and Indian children occupy significantly more time on mobile device activity compared to Chinese children [110]. Therefore, future studies may address the differences in the way parents provide guidance and monitor their children’s mobile device use. The positive aspect of guiding and monitoring could be useful and applicable for other parents.

Secondly, regarding the validity and reliability of the items in the adapted version of the Problematic Mobile Phone Use Scale (PMPUS). This research examines the validity and reliability of the items by performing exploratory factor analysis (EFA) and determining the value of the content validity index and Cronbach’s alpha. Therefore, a future study can broaden the item assessment by performing Rasch analysis and confirmatory factor analysis (CFA). Rasch analysis is an advanced approach used to enhance the accuracy of instruments development, monitor instrument quality, and compute participants’ performances [111]. Meanwhile, confirmatory factor analysis was used to verify the number of underlying dimensions of the instrument and the pattern of item–factor relationships. Rasch analysis will provide comprehensive information on items properties, and confirmatory factor analysis will reveal to what extent the subdimension measures the variables.

Thirdly, this study employed a cross-sectional research design, and data were collected using a questionnaire. Although the data collected are credible to explain the causal relationship between the variables, the data may not be able to explain the reason for such a relationship existing [112]. This issue can be overcome by employing a mixed-method research design and collecting quantitative and qualitative data. Qualitative data from interviews and observation may help in providing useful information and explanation of the effects of sociodemographic factors on mobile device usage.

Lastly, this study only examines the direct relationship between sociodemographic factors and excessive mobile device use of young children. However, future study may include the potential mediator variables in order to provide more meaningful findings. The mediator variables will reveal how the mediator affects the strength of the relationship between sociodemographic factors and excessive mobile device use. The potential mediator variable that can be applied is the period of using a mobile device.

## 6. Conclusions

This study investigated the category of mobile device users among young children in Putrajaya, Malaysia. In addition, this study also examined the influence of sociodemographic factors, namely, gender, the child’s age when starting to use a mobile device, parent’s level of education, monthly household income, frequent types of applications, and the purpose of giving mobile devices to young children. The findings of this study show that the majority of participants were regular users, followed by the at-risk user, casual user, and problematic user. Analysis of the influences of sociodemographic factors revealed that gender, age of children when starting to use mobile devices, and the purpose of providing mobile devices to children contributed significantly to excessive mobile use by young children. Based on the findings of this study, parents should create a healthy environment on a mobile device by providing guidance and monitoring the duration and content of the applications used by children. Next, these sociodemographic factors can be taken into account when planning for intervention or guidance programs on mobile device use, especially for young children.

## Figures and Tables

**Table 1:** Demographic information on the children and their families.

Variables	Categories	*n* (%)
Children’s Characteristics		
Gender	Male	176 (48.4)
	Female	188 (51.6)
Age (years)	5	186 (51.1)
	6	178 (48.9)
Family Characteristics		
Age (years)	25 and below	3 (0.8)
	26–35	167 (45.9)
	36 and above	194 (53.3)
Household income	T20	121 (33.2)
	M40	131 (36.0)
	B40	112 (30.8)
Level of education	Certification	91 (25.0)
	Diploma	78 (21.4)
	Bachelor’s degree	130 (35.7)
	Master’s/doctoral degree	65 (17.9)

**Table 2:** Mobile device usage information.

Variables	Categories	*n* (%)
Types of device	Smartphone	265 (72.8)
	Tablet	99 (27.2)
Age at start of use (years)	3 and below	303 (83.2)
	4–6	61 (16.8)
Type of application	Education	71 (19.5)
	Entertainment	293 (80.5)
Ownership	Self-owned	34 (9.3)
	Parents	287 (78.8)
	Family members	43 (11.8)
The purpose of providing a mobile device	Sit still	119 (32.7)
	Tantrums	39 (10.7)
	Education	124 (34.1)
	Technology updates	82 (22.5)

**Table 3:** User categories and scores ranges.

User Categories	Percentile	Score Ranges
Casual user	Below 15th	18–24
Regular user	15th to below 80th	25–45
At-risk user	80th to below 95th	46–55
Problematic user	95th or above	56–90

**Table 4:** Descriptive analysis and internal consistency of items via Cronbach’s alpha coefficients.

Items	*M*	*SD*	Cronbach’s Alpha Value if the Item is Removed
D1	2.286	0.982	0.904
D2	2.176	0.957	0.901
D3	2.258	0.939	0.903
D5	2.211	0.879	0.901
D6	1.550	0.761	0.899
AO10	2.160	0.997	0.905
AO11	1.830	0.911	0.905
AO12	1.717	0.833	0.902
AO13	1.750	0.772	0.898
AO15	1.945	0.904	0.906
CP16	2.264	0.969	0.909
CP17	2.431	1.028	0.899
CP19	1.876	0.874	0.900
CP20	2.586	1.026	0.912
IA22	1.931	0.752	0.905
IA23	1.631	0.678	0.907
IA24	2.184	0.931	0.909
IA25	1.720	0.730	0.907

*M* = mean; *SD* = standard deviation.

**Table 5:** Factor loading from the principal axis factor analysis with varimax rotation for a four-factor solution and adapted PMPUS (*n* = 364).

Items	Factor Loading				Communality
	1	2	3	4	
AO12	0.764				0.657
AO13	0.738				0.659
AO11	0.733				0.631
AO10	0.525				0.525
AO15	0.443				0.480
D2		0.792			0.720
D3		0.760			0.653
D1		0.756			0.652
D6		0.588			0.510
D5		0.573			0.507
IA22			0.754		0.691
IA25			0.656		0.517
IA23			0.628		0.557
IA24			0.494		0.390
CP17				0.549	0.368
CP16				0.475	0.364
CP20				0.459	0.382
CP19				0.449	0.400
Eigenvalues	2.962	2.611	2.360	1.729	
% of variance	16.457	14.504	13.109	9.603	

**Table 6:** Percentage of users based on category.

User Categories	*n*	%	*M*	*SD*
Casual users	54	14.8	21.52	2.13
Regular users	234	64.3	35.46	5.33
At-risk users	64	17.6	48.59	2.34
Problematic users	12	3.3	59.50	2.72

*M* = mean; *SD* = standard deviation.

**Table 7:** Binary logistic regression analysis for potential factors for problematic users.

Independent Variables	β	SE	Wald	df	*p*	Odds Ratio	95% for OR	
							Lower	Upper
Gender	−0.755	0.280	7.288	1	0.007 **	0.470	0.272	0.813
Age	−1.341	0.500	7.179	1	0.007 **	0.262	0.098	0.698
Education			2.530	3	0.470			
Certification	−0.568	0.401	2.011		0.156	0.567	0.258	1.242
Diploma	−0.547	0.423	1.672		0.196	0.579	0.252	1.326
Bachelor’s	−0.362	0.470	0.592		0.442	0.696	0.277	1.750
Income			1.822	2	0.402			
B40	−0.497	0.371	1.791		1.181	0.608	0.294	1.260
M40	−0.278	0.432	0.413		0.520	0.758	0.325	1.767
Types of	0.114	0.319	0.127	1	0.721	1.121	0.600	2.094
Application	0.206	0.366	0.318	1	0.573	1.229	0.600	2.517
Purpose			10.334	3	0.016 **			
Tantrums	1.142	0.428	7.122		0.008 **	3.1354	1.354	7.254
Education	−0.237	0.350	0.456		0.499	0.789	0.397	1.569
Technology	0.80	0.376	0.045		0.832	1.083	0.519	2.260
Constant	−1.141	0.705	0.040		0.841	0.868		

## Data Availability

The data presented in this study are available upon request from the corresponding author. The data are not publicly available due to the risk of the identification of study participants.

## Appendix A

**Table A1:** Adapted Version of PMPUS.

D1. My child will be restless if the mobile device he is using does not have an internet connection
D2. My child will be restless if the mobile device he is using runs out of battery
D3. My child will be agitated if the mobile device he is using lags
D5. My child seems unhappy if not given a mobile device
D6. My child finds it difficult to sleep if not given a mobile device
AO10. My child is busy using mobile devices that it interrupts his mealtime routine
AO11. My child is busy using mobile devices that interferes with his sleep routine
AO12. My child is busy using mobile devices that he has trouble completing preschool homework
AO13. My child is busy using mobile devices that he has trouble focusing on learning
AO15. My child is busy using mobile devices that it affects his interaction with the people around him
CP16. My child would have a tantrum if the mobile device he was using was taken away
CP17. My child is using a mobile device beyond the set period
CP19. My child uses a mobile device for more than two hours a day
CP20. My child will look for a mobile device as soon as he wakes up
IA22. My child would rather spend time with a mobile device than hang out with the people around him
IA23. My child prefers to have conversations using a mobile device, rather than face to face
IA24. My child prefers to use a mobile device alone
IA25. My child prefers to play game using a mobile device than play with friends
